# Malignant Gastrointestinal Stromal Tumor of Rectum: A Case Report and Review of Literature

**DOI:** 10.1055/s-0042-1742778

**Published:** 2022-02-16

**Authors:** Mohan Karthikeyan, Chinnusamy Kolandasamy, Obla L. Naganath Babu

**Affiliations:** 1 Institute of Surgical Gastroenterology, Madras Medical College and Rajiv Gandhi Government General Hospital, Chennai, Tamil Nadu, India

**Keywords:** gastrointestinal stromal tumor, rectum, laparoscopic resection, immunohistochemistry, high risk, imatinib

## Abstract

Gastrointestinal stromal tumors (GISTs) are rare tumors of the gastrointestinal tract accounting for less than 1% of all gut tumors. GISTs occurring in the rectum are extremely rare and these usually present at an advanced stage compared with other sites.

We report a case of a middle-aged female who presented with features of anemia and subacute obstruction due to a large rectal tumor and underwent abdominoperineal resection. The histopathological examination confirmed the diagnosis of high-grade malignant GIST with multiple lymph nodal metastasis. She was started on adjuvant imatinib therapy and is on follow-up without any evidence of recurrence.

The authors conclude that GIST must be included in the differential diagnosis of a rectal tumor. Diagnosis is established by biopsy and immunohistochemistry studies. Surgical resection with histological negative margins is the standard curative treatment. Adjuvant targeted therapy can reduce long-term recurrence in high-risk cases.

Gastrointestinal stromal tumors (GISTs) are the most common mesenchymal tumors involving the gastrointestinal tract. These tumors most commonly occur in sixth and seventh decades of life. The most common sites involved are the stomach (50–65%) followed by the small intestine (20–30%). 1 Metastatic spread commonly occurs to the liver and the peritoneum. Lymph node metastasis is very uncommon. Rectal GISTs are extremely rare. These usually present at an advanced stage and have poor prognosis compared with tumors at other sites. Here, we present a case of a large malignant GIST of rectum managed by laparoscopic resection.

## Case Report

A 40-year-old female was referred to our department with complaints of bleeding per rectum and mass descending per rectum for 2 months and with recent onset lower abdominal pain. She was anemic at presentation. Clinical examination of the abdomen did not reveal any abnormality. Digital rectal examination revealed an eccentric proliferative friable growth, fixed at its base, starting at 3 cm from anal verge occupying almost the entire lumen. The upper limit of the lesion could not be reached. Blood staining was detected on the gloved finger. There was no inguinal lymphadenopathy.

Proctoscopy reveled an intraluminal near-circumferential proliferative growth starting at the level of dentate line, extending upwards into the rectum, and the surface was covered with fresh and altered blood. Its upper extent could not be visualized. A provisional diagnosis of rectal malignancy was made. Laboratory investigations revealed microcytic hypochromic anemia (hemoglobin = 5.5 g%) and normal CEA (carcinoembryonic antigen) levels.

**Fig. 1:**
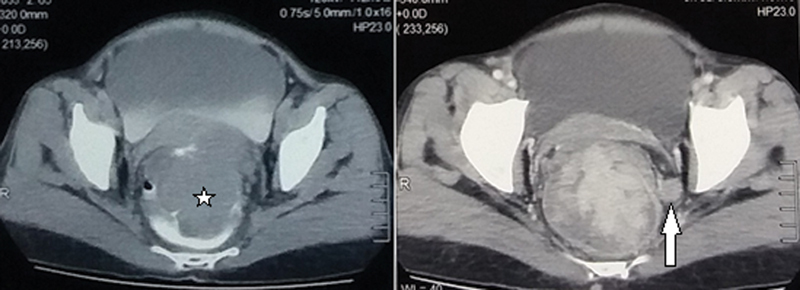
Axial sections of CT pelvis showing large lobulated enhancing intraluminal mass (★) with enlarged perirectal nodes (arrow). CT, computed tomography.

Contrast-enhanced computed tomography (CT) of the abdomen which was done elsewhere, showed an irregular polypoidal wall thickening showing contrast enhancement, extending from just above the anal verge up to the rectosigmoid junction, with surrounding mesorectal fat stranding and multiple enlarged perirectal nodes ( Fig. 1 ) *.*

**Fig. 2:**
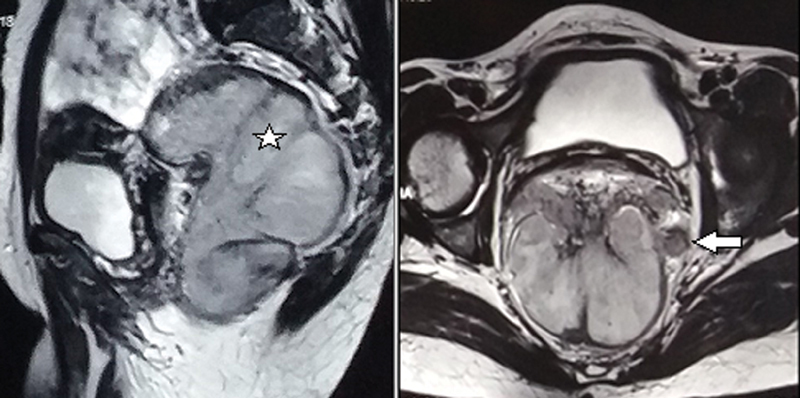
Sagittal and axial sections of MRI pelvis showing a large lobulated mass (★) almost occupying the entire rectal lumen with multiple enlarged perirectal nodes (arrow). MRI, magnetic resonance imaging.

Magnetic resonance imaging (MRI) of the pelvis was done in our institution. It showed a diffuse polypoidal endoluminal lesion extending from 3.5 cm above anal verge up to rectosigmoid junction measuring approximately 14-cm long with infiltration into mesorectal fat, thickening of mesorectal fascia, anal sphincter involvement and multiple enlarged perirectal and bilateral internal iliac nodes ( Fig. 2 ).

Proctoscopy-guided biopsy of the lesion was reported as amelanotic type of malignant melanoma. Sigmoidoscopy could not be performed due to large size of the growth. CT scans of the abdomen and chest did not reveal any distant metastasis.

**Fig. 3:**
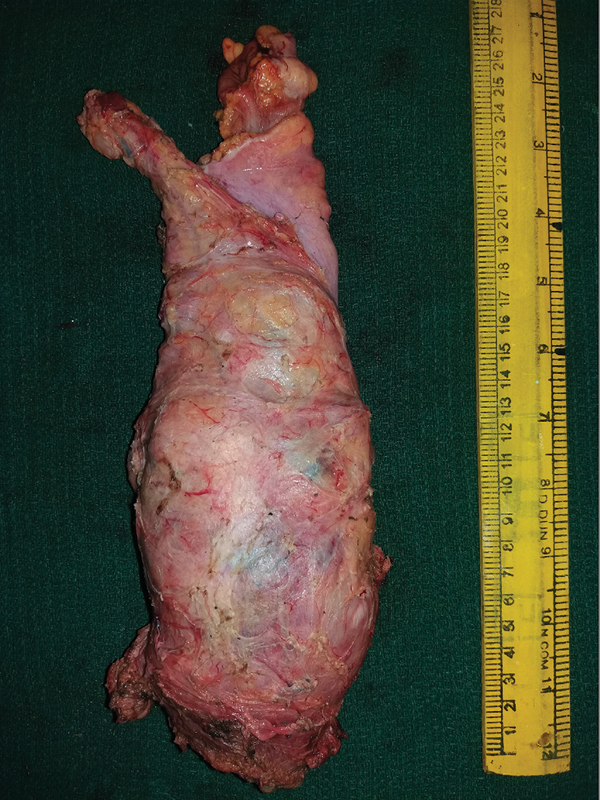
Resected specimen, abdominoperineal excision.

The patient was optimized with blood transfusion and intravenous fluids with electrolyte supplementation. Since she had persistent bleeding from the tumor and had features suggestive of intermittent subacute intestinal obstruction, she was taken up for surgery as per the opinion of the institutional multidisciplinary tumor board and underwent laparoscopic abdominoperineal excision (APE) of rectum ( Fig. 3 ).

**Fig. 4:**
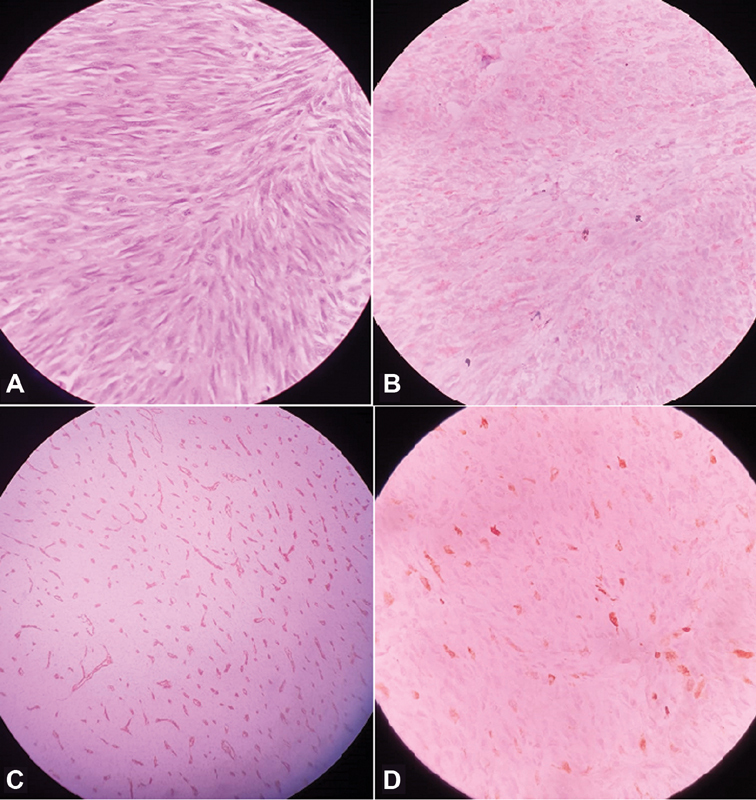
HPE and IHC study ( **A** ) High power view showing spindle shaped malignant cells arranged in fascicles, ( **B** ) CD 117 positive, ( **C** ) CD 34 positive, and ( **D** ) S-100 negative. HPE, histopathological examination; IHC, immunohistochemistry.

The histopathological examination (HPE) showed a 11-cm pedunculated proliferating mass formed by of a neoplasm composed of spindle shaped cells arranged in fascicles and bundles, and involving up to serosa of the bowel wall and anal sphincters, with mitotic count of 25/50 high power fields (HPF), and all 14 lymph nodes harvested showed metastatic deposits. Diagnosis of GIST was confirmed by immunohistochemistry (IHC) which was positive for CD 117 and CD 34, and negative for CK (cytokeratin), S-100, HMB 45 (human melanoma black 45), and α-SMA (smooth muscle actin; Fig. 4 ) *.*

A final diagnosis of malignant GIST of the rectum stage IV (T _4_ N _1_ M _0_ ), as per the American Joint Committee on Cancer (AJCC) staging system, was made. Postoperative period was uneventful. She recovered well and was discharged on postoperative day 11. She was started on imatinib adjuvant therapy (400-mg daily). She is on regular follow-up for the past 30 months, with contrast-enhanced CT of abdomen and pelvis done once every 6 months, without any evidence of recurrence.

## Discussion

Gastrointestinal stromal tumors (GISTs) originate from the interstitial cells of Cajal and stain positive for CD117, a product of c-kit protooncogene involved in the regulation of cell proliferation. Majority of GISTs develop due activating mutations in the c-kit or the platelet-derived growth factor receptor A ( *PDGFRA* ) genes leading to unchecked cellular proliferation. 2

About 5% of all GISTs occur in the rectum and they account for 0.1% of all tumors originating in the rectum. 3 Only three cases of rectal GIST were treated at our institution over a 3-year period from 2017 to 2020, including our patient. These tumors present with bleeding per rectum, lower abdominal pain, tenesmus, constipation, urinary symptoms, or may be occasionally asymptomatic.

Most tumors originate within the muscularis propria and commonly have an exophytic growth pattern. On sigmoidoscopy, rectal GIST can be seen as an intraluminal or a submucosal mass. Multimodal imaging with CT, MRI, and endoscopic ultrasound (EUS) is helpful in evaluation of tumor size, morphology, depth of infiltration, and metastasis. They appear as hypervascular, enhancing masses, and are often heterogeneous due to areas of hemorrhage, necrosis, or cystic degeneration. 4 These tumors commonly present with distant metastasis (47%). 2 Positron emission tomography (PET) is useful for detection of metastasis and for assessing response to medical therapy. 5 6 As per NCCN guidelines, PET is not routinely recommended for staging except for clarification of ambiguous findings on cross-sectional imaging and when neoadjuvant therapy is planned. 7 PET was not done in our case. Instead, staging laparoscopy was performed at the time of definitive surgery to rule out peritoneal metastasis.

Biopsy with immunohistochemical analysis is crucial for accurate diagnosis, for initiating neoadjuvant therapy, and for surgical planning. Histologically, these tumors may exhibit a spindle pattern, an epithelioid pattern, or a mixed subtype. Others tumors that can mimic GIST on histology include leiomyoma, leiomyosarcoma, desmoid, neuroendocrine tumor, fibrous tumors, melanoma, and other sarcomas. 8 Positive expression of CD117 (c-kit) is the major diagnostic criteria with high sensitivity (95%). 9 Rectal GISTs also show high incidence of CD34 positivity (90%). 10 (Detected on GIST-1 (DOG1) is a recently identified marker with sensitivity similar to CD117 in the diagnosis of these tumors, including wild-type GISTs which have no detectable c-kit or PDGFRA mutations and constitute 10 to 15% of all GISTs. DOG1 immunostaining is useful in cases that cannot be categorized as GIST based on CD117 immunostaining and mutation testing for KIT and PDGFRA. 7 11 In our case, preoperative biopsy was reported as amelanotic melanoma but IHC study was not done.

Complete surgical resection with histologically negative margins (R _0_ resection) is the standard curative procedure for localized tumors and is essential to prevent recurrence. Care must be taken to avoid intraoperative tumor rupture. As these tumors do not spread through lymphatics, routine lymph node dissection is unnecessary except if lymph node involvement is suspected during surgery. The incidence of lymph-node metastasis in GISTs overall has been reported to be around 1%. 12 The prognostic significance of nodal metastasis has not been clearly established. Our patient had extensive pelvic lymph node metastasis which has been reported rarely in the literature.

The choice of surgical procedure (local excision, anterior resection, and abdominoperineal excision of rectum) depends on tumor size and location. Whenever feasible, sphincter-preserving resection is recommended. If Abdominoperineal excision of rectum is needed to achieve margin negative resection, then preoperative chemotherapy is recommended. 13 The anus-preservation rate following chemotherapy has been reported to range from 33 to 94.9%. 14 Several case reports have demonstrated that use of preoperative (neoadjuvant) imatinib enables sphincter-sparing resection and improves survival for rectal GISTs. 15 Since preoperative biopsy was reported as melanoma in our case, upfront resection was performed. Even if it had been reported as GIST, upfront resection could be justified due to its large size causing luminal obstruction and presence of persistent bleeding from the tumor. There are similar case reports of large anorectal GISTs managed by abdominoperineal excision. 16 17 18

Laparoscopic resection has been successfully performed for rectal GISTs. 14 19 Laparoscopic APE had to be done in our case due to extensive sphincter involvement as per imaging and ongoing bleeding from tumor. Transanal endoscopic microsurgery (TEM) and transanal minimally invasive surgery (TAMIS) have also been performed for rectal GISTs with good success. 20

About 40 to 50% of GISTs will recur or develop metastasis even after a curative resection. Criteria, such as the Fletcher criteria, Armed Forces Institute of Pathology (AFIP) criteria, and Joensuu criteria, have been developed to predict the risk of disease recurrence. These are based on factors such as tumor size, mitotic count, tumor location, and presence of tumor rupture. Those who are in high-risk category benefit from adjuvant therapy. Rectal GISTs tend to have aggressive biological behavior and tumors with high mitotic activity can recur and metastasize despite a small size of <2 cm. Predicted metastasis rate in our case as per AFIP criteria was 71 to 90% and belonged to high-risk category. 9 21 22

Adjuvant therapies using small-molecule tyrosine kinase inhibitors, imatinib mesylate and sunitinib, have demonstrated good results in improving survival. These drugs act by selectively blocking c-kit function and thereby halting proliferation. Those with c-kit mutation at exon-11, which has been reported commonly in rectal GISTs, respond better to treatment than those with mutation at exon-9. Thus, c-kit mutation genotype analysis is essential before starting therapy whenever feasible. 23 In a landmark trial by DeMatteo et al, the use of imatinib in the adjuvant setting was shown to be associated with improvement in survival. They reported 5-year overall survival rate of 83% and recurrence-free survival rate of 40%. 24 There is no definite consensus on the optimal duration of adjuvant therapy but is generally recommended for a period of 3 years. 25 26

Tumor size, mitotic index, R _0_ resection, and c-kit positivity are important prognostic factors. 2 3 Benign GISTs have no evidence of local invasion and have low mitotic activity, and thus have favorable prognosis with local excision alone. Malignant GISTs are locally invasive and/or metastatic at presentation, or recur after resection. These tumors are usually large (>5 cm) and have a high mitotic count (>5/50 HPF). 27 Patients with malignant GISTs have an overall 5-year survival rate of approximately 45%. 2 The recurrence rate has been shown to be as high as 40% even after early resection. 2 21 Majority of recurrences occur either locally or in the liver. Hence, long-term follow-up with 3 to 6 monthly imaging for 3 to 5 years and then annually is generally recommended.

## Conclusion

Gastrointestinal stromal tumors are rare malignancies involving the rectum. Diagnosis is established by biopsy and immunohistochemistry. Lymph node metastasis is very rare. Complete surgical resection with negative margins is the treatment of choice. The appropriate surgical technique should be selected based on location, size, and resectability of tumor and the available surgical expertise. Adjuvant imatinib therapy prolongs survival in high-risk tumors.

This case is reported for its rarity and presentation with lymph nodal metastasis.
